# Detecting delirium in Parkinson’s disease: an evaluation of diagnostic accuracy of bedside tools

**DOI:** 10.1093/ageing/afaf197

**Published:** 2025-07-20

**Authors:** Rachael Ashleigh Lawson, Sarah Joanna Richardson, Florence Gerakios, Alison Jane Yarnall, Gemma Bate, Laura Wright, Claire McDonald, John Paul Taylor, David Burn, Glenn Stebbins, Louise M Allan

**Affiliations:** Translational and Clinical Research Institute, Newcastle University, Newcastle upon Tyne, England, United Kingdom of Great Britain and Northern Ireland; NIHR Newcastle Biomedical Research Centre, Newcastle upon Tyne, England, United Kingdom of Great Britain and Northern Ireland; Newcastle upon Tyne Hospitals NHS Foundation Trust, Newcastle upon Tyne, England, United Kingdom of Great Britain and Northern Ireland; Translational and Clinical Research Institute, Newcastle University, Newcastle upon Tyne, England, United Kingdom of Great Britain and Northern Ireland; NIHR Newcastle Biomedical Research Centre, Newcastle upon Tyne, England, United Kingdom of Great Britain and Northern Ireland; Translational and Clinical Research Institute, Newcastle University, Newcastle upon Tyne, England, United Kingdom of Great Britain and Northern Ireland; NIHR Newcastle Biomedical Research Centre, Newcastle upon Tyne, England, United Kingdom of Great Britain and Northern Ireland; Newcastle upon Tyne Hospitals NHS Foundation Trust, Newcastle upon Tyne, England, United Kingdom of Great Britain and Northern Ireland; Translational and Clinical Research Institute, Newcastle University, Newcastle upon Tyne, England, United Kingdom of Great Britain and Northern Ireland; NIHR Newcastle Biomedical Research Centre, Newcastle upon Tyne, England, United Kingdom of Great Britain and Northern Ireland; Newcastle upon Tyne Hospitals NHS Foundation Trust, Newcastle upon Tyne, England, United Kingdom of Great Britain and Northern Ireland; Translational and Clinical Research Institute, Newcastle University, Newcastle upon Tyne, England, United Kingdom of Great Britain and Northern Ireland; NIHR Newcastle Biomedical Research Centre, Newcastle upon Tyne, England, United Kingdom of Great Britain and Northern Ireland; NIHR Newcastle Biomedical Research Centre, Newcastle upon Tyne, England, United Kingdom of Great Britain and Northern Ireland; Newcastle upon Tyne Hospitals NHS Foundation Trust, Newcastle upon Tyne, England, United Kingdom of Great Britain and Northern Ireland; Translational and Clinical Research Institute, Newcastle University, Newcastle upon Tyne, England, United Kingdom of Great Britain and Northern Ireland; NIHR Newcastle Biomedical Research Centre, Newcastle upon Tyne, England, United Kingdom of Great Britain and Northern Ireland; Gateshead Health NHS Foundation Trust, Gateshead, England, United Kingdom of Great Britain and Northern Ireland; Translational and Clinical Research Institute, Newcastle University, Newcastle upon Tyne, England, United Kingdom of Great Britain and Northern Ireland; NIHR Newcastle Biomedical Research Centre, Newcastle upon Tyne, England, United Kingdom of Great Britain and Northern Ireland; Translational and Clinical Research Institute, Newcastle University, Newcastle upon Tyne, England, United Kingdom of Great Britain and Northern Ireland; Department of Neurological Sciences, Rush University Medical Center, Chicago, Illinois, United States; Centre for Research in Ageing and Cognitive Health, University of Exeter, Exeter, England, United Kingdom of Great Britain and Northern Ireland

**Keywords:** Parkinson’s disease, delirium, diagnosis, cognitive impairment, dementia, older people

## Abstract

**Background:**

Delirium is a serious, acute neuropsychiatric condition associated with fluctuating attention and altered arousal. Delirium in Parkinson’s disease (PD) is common but often missed in hospital due to shared clinical features. This study aimed to evaluate the accuracy of current tools used to identify delirium in inpatients with PD.

**Methods:**

People with PD admitted to all hospital wards were invited to take part. Participants completed a standardised delirium assessment based on the Diagnostic and Statistical Manual of Mental Disorders 5th Edition (DSM-5) criteria, in addition to standard bedside tools including the 4 As Test (4AT), arousal and cognition. This was a secondary analysis of a prospective observational study; bedside tools were not completed independently of, or blinded to, the DSM-5 criteria. Accuracy was assessed using Receiver Operating Characteristic area under the curve (AUROC).

**Results:**

Participants included 115 people with PD (200 hospital admissions); 66.1% (n = 76/115) had delirium. Considering all admissions, the diagnostic accuracy of tools was good, ranging from 74% to 89% (AUROC = 0.764-0.923, *P* < .001 for all). The 4AT scores had the highest sensitivity (96.7%, AUROC = 0.922, *P* < .001). However, accuracy decreased in those with underlying cognitive impairment (AUROC = 0.499–0.886).

**Conclusions:**

Current bedside tools can accurately identify delirium in PD inpatients. Although tools were comparable, the 4AT may have greater clinical utility as it had high sensitivity, is quicker to complete and already widely used clinical. However, caution is recommended as tools did not differentiate between symptoms typical in PD and acute symptoms associated with delirium; this should be a focus for future research.

## Key Points

- Delirium in Parkinson’s disease (PD) is common but often missed.
- We found that most assessments have good diagnostic accuracy in PD.
- Although the bedside diagnostic tools were comparable, the Four As Test may have greater clinical utility as it is quicker to complete and already widely used clinically.
- The accuracy of all tools was lower in PD participants with known cognitive impairment, and different cut-offs may be needed to aid delirium diagnosis.
- The findings from this study will help clinical teams to choose appropriate tools to identify delirium accurately in PD, leading to improved patient outcomes.

## Introduction

Delirium is a serious, neuropsychiatric syndrome defined by acute changes in attention, level of arousal and cognition [1]. It is distressing to patients and their families, associated with high healthcare costs, poor outcomes and cognitive decline [2, 3]. Delirium is often both underreported and under recognised, resulting in worse patient outcomes [4, 5]. People with Parkinson’s disease (PD) are at increased risk of developing a delirium in hospital, with 66.9% of PD participants identified as having delirium compared to 38.7% of older adults without PD [6], and have an increased risk of death, institutionalisation and developing a new dementia in 12 months compared to those without delirium.

Delirium in PD is poorly recognised as it can be challenging to identify due to shared clinical features, such as cognitive fluctuations, inattention, visual hallucinations, delusions and disrupted sleep [7, 8]. Previous studies are limited by inconsistent delirium ascertainment or reliance on retrospective diagnoses [7, 9]. Commonly used delirium tools such as the Four As Test (4AT) [10] and the Confusion Assessment Method (CAM) [11] have been applied in PD, but their accuracy has not been evaluated [9]. A small study (n = 54) evaluating a limited selection of bedside tools found that arousal tools were reported to have a good overall accuracy in PD, but attention tests performed poorly in those with Lewy body cognitive impairment [12]. This is contrary to the wider delirium literature and suggests the need for further detailed phenomenological study of delirium specifically in people with PD [13].

This study aimed to evaluate the diagnostic accuracy using a secondary analysis of a range of commonly used bedside tools comprising delirium screening, delirium severity and brief cognitive tools across cognitive domains in inpatients with PD and those with PD with known cognitive impairment.

## Methods

### Population

Participants were from the ‘Defining Delirium and its Impact in Parkinson’s Disease’ (DELIRIUM-PD) study [6]. The DELIRIUM-PD study is a prospective observational study which included patients who attend outpatient clinics in Newcastle Hospitals for the management of their PD and were admitted to hospital between 7^th^ March 2018 and 31st January 2022 (the study was paused due to the COVID-19 pandemic for a period of 10 months). This study was approved by Research Ethics Committees (18/YH/0486).

Recruitment methods have previously been described [6]. In brief, potential participants were provided with written information with contact details to opt out of further contact (Supplementary Figure 1). An electronic recurring admission patient alert was added to their records to alert researchers when they were admitted to hospitals in Newcastle upon Tyne, UK. The research team aimed to approach participants within 72 hours of admission to invite them to participate, written informed consent was then sought. This included both elective and non-elective admissions. Next of kin or appropriate relative/friend were also invited to take part as informants and written informed consent was sought.

A formal capacity assessment based on the Mental Capacity Act [14] was performed by a trained member of the research team. If participants lacked capacity to provide full written informed consent, a personal consultee was identified. They provided advice on participation as per Section 32 of the Mental Capacity Act.

Exclusion criteria comprised a diagnosis of non-idiopathic PD or atypical parkinsonian disorders, lacked capacity to give informed consent and did not have an appropriate consultee available, were receiving end of life care, isolated for infection control, or had insufficient command of the English language to take part in the cognitive assessments.

### Data collection

Baseline demographic and clinical data were collected at recruitment, including age, sex, comorbidities, medications and frailty (using the Clinical Frailty Scale, CFS, score range 0-9, higher scores indicating greater frailty) [15]. Participants with baseline mild cognitive impairment (PD-MCI) or PD dementia (PDD) were identified from clinic letters by their treating clinician. PD duration, levodopa equivalent daily dose (LEDD) [16], motor severity as measured by the total Movement Disorder Society – Unified Parkinson’s Disease Rating Scale Part III (MDS-UPDRS III) score (score range 0–132, higher scores indicating greater motor severity) and the Hoehn and Yahr stage (functional disability of PD, ranging from 0–5; higher stage indicated greater disability) were also assessed [16].

#### Delirium ascertainment

Delirium was systematically assessed prospectively using a structured interview based upon the Diagnostic and Statistical Manual of Mental Disorders 5^th^ Edition (DSM-5) criteria [1] as described previously by Richardson, *et al.* [3] (Supplementary Table 1) and were used as our reference standard. In brief, this comprised researcher observations, standardised testing and information obtained from the informant and hospital staff to establish an acute change from baseline. When there was diagnostic uncertainty, vignettes were presented to an expert consensus panel made up of two expert independent reviewers and a third for any disagreement of cases (FG and RAL, and AJY or SJR) [17]. Delirium assessments were performed consecutively for up to five days where possible. Following this, participants were seen once weekly until discharge, and on all subsequent admissions during the recruitment period.

At each visit, we evaluated the performance of commonly used delirium bedside tools (Table 1). Where there was overlap in assessments (e.g. orientation, digit span, disorganised thinking), performance as part of the DSM-5 criteria were used to rate items (e.g. CAM, 4AT, DSR-98-R). As this was a secondary analysis of a prospective observational study, bedside tools were not completed independently of, or blinded to, the DSM-5 criteria. Where participants had altered levels of attention and arousal where they were unable to answer to questions (i.e. severe hypo- or hyperactivity), poorest scores were given and informants were used to rate symptoms (e.g. hallucinations, delusions).

**Table 1:** Scoring of bedside delirium tool recorded for all participants.

Bedside tool	Description	Scoring used
MDAS total [18]	10 item assessment measuring delirium severity	Each item scored 0–3, total score range 0-30, score of ≥12 indicates delirium
DSR-98-R total [19]	13 item assessment measuring delirium severity	Each item scored 0–3, total score range 0-39, score of ≥15 indicates delirium
4AT total [10]	Brief screening tool with 4 domains capturing attention, arousal, acute onset and fluctuating course	Total score range 0–12, score of ≥4 indicates delirium
CAM [11]	Brief screening tool with 4 domains capturing acute onset and fluctuating course, inattention, disorganised thinking and altered consciousness	Delirium present/absent if meet two core criteria and one of two supporting criteria
SQuID [20]	Carers asked if participant seems more confused than usual	Yes/No
**Attention**		
Digits forward [13]	Participant asked to repeat a string of numbers in the same order.	Number of digits correctly repeated, score range 0–5
Digits backwards [13]	Participant asked to repeat a string of numbers in reverse order	Number of digits correctly repeated, score range 0–4
Months of the year backwards [10]	Participant asked to recite the months of the years in backwards order	0 = ≥7 months recalled, 1 = refused/<7months correct, 2 = untestable
SAVEAHAART [13]	Letter vigilance test, patient taps hand on each letter A	Impaired: >1 error
20to1 [13]	Participant counts down from 20 to 1	Impaired: >1 error
Serial 7s [13]	Participant asked to subtract 7 from 100 five times	Number correct, score range 0–5
Trail making Part A^*^	Participant asked to join the numbers from 0-20 sequentially as fast as possible.	Time taken to complete test in seconds, range 0–300 seconds
**Arousal**		
GCS [21]	Observational assessment capturing three domains: eye response, voice response, movement response	Total score range 0–15, lower scores indicating poorer score
OSLA [22]	Observational assessment capturing 4 domains: eye opening, eye contact, posture, movement	Total score range 0–15, with higher scores indicating greater derangement in level of arousal
m-RASS [23]	Single item observational assessment capturing increase or reduced arousal.	Scores range from +4 to -5; normal score = 0
**Cognition**		
***Orientation***		
Orientation total	10 item orientation question from the MMSE	Number correct, score range 0–10
Age	Participant asked their age	Correct/incorrect
***Memory***		
Date of birth	Participant asked their date of birth	Correct/incorrect
WWII end	Participant asked which year World Ward II ended	Correct/incorrect
Three-word immediate recall	Participant given three words to immediately recall.	Number of words correctly recalled on first attempt, score range 0–3
Delayed recall	Participants asked to recall three words after five minutes	Number of correctly words recalled after five minutes, score range 0–3
***Visuospatial***		
Stone float on water	Participant asked, “Will a stone float on water?”	Correct/incorrect
***Language***		
Object naming	Participant shown a pen, asked to name the object and describe its use	One point for each correct answer, score range 0–2
Three stage command	Participant asked to take pen in right hand, touch their nose and give back	Point given for each command correctly followed, score range 0–3

^*^ *Missing data in n = 46 admissions*

*MDAS: Memorial Delirium Assessment Scale, 4AT:4 As Test, CAM: Confusion Assessment Method Supplementary Table 2, DSR-98-R: Delirium Rating Scale, SQuID: Single Question in Delirium; GCS* = *Glasgow Coma Scale; OSLA* = *Observational Level of Arousal; m-RASS* = *Modified Richmond Agitation Scale, WWII: World War II, MMSE* = *Mini Mental State Examination.*

### Statistical analysis

Statistical analyses were performed using SPSS software (Version 28.0; SPSS, Armonk, NY: IBM Corp). We used MDAS to identify peak delirium severity in all admissions of all participants; the study visit identified as peak delirium severity was used in the subsequent analysis. Data were examined for normality of distribution with visual histograms and Kolmogorov-Smirnov’s test. Comparisons between two groups were performed using independent t-tests, Mann-Whitney U tests or Pearson χ2 tests, as appropriate, between participants with and without delirium. For the purposes of analysis, each admission was included as a separate case.

The accuracy of the tools was determined using the area under the receiver operating characteristic (ROC) curves (AUC) against the gold standard DSM-5 criteria for delirium. Paired sample area difference of AUC was used to compare tool accuracy. Sensitivity and specificity scores were calculated and Youden’s index was used to identify optimal cut-offs. To aid interpretation, the m-RASS was transformed so that negative scores were multiplied by -1, thus all scores were positive (0-5). To evaluate the assessments in those with cognitive impairment, analysis was repeated in participants with known baseline cognitive impairment (PD-MCI or PDD). For all analyses, we applied Benjamini-Hochberg multiple comparisons correction with a 5% false discovery rate.

## Results

We recruited 115 participants with PD during the study period, accounting for 200 individual hospital admissions (Supplementary Figure 1). Of these, 66.1% (n = 78) had delirium during the study period (Table 2). Participants with delirium were significantly older, frailer, had greater motor severity, more likely to have baseline cognitive impairment and less functional independence (*P* < .05 for all). There were no other significant differences (*P* > .05 for all). Participants with delirium had a greater number of admissions (median, interquartile range [IQR] = 1, 2 vs. 1, 0, respectively, *P* < .05) and study visits (median, IQR = 6, 8 vs. 4.5, 5, respectively, *P* < .05) compared to those without delirium.

**Table 2:** Characteristics of study participants.

		PD no delirium	PD with delirium		Test statistic	p-value
		n = 39	n = 76			
Age (years)		75(18)	77(10)	Z	-2.0	**0.044**
Sex: female n,%		17(43.6)	28(36.8)	X^2^	0.5	0.483
Education (years)		11(3)	11(3)	Z	-0.5	0.640
PD duration from diagnosis (months)		97(109)	69(82.8)	Z	-1.8	0.077
PD duration from diagnosis (years)		8.1(9.1)	5.8(6.9)	Z	-1.8	0.077
MDS-UPDRS III total^*^		50(13)	62.5(25)	Z	-2.9	**0.003**
Hoehn and Yahr stage^†^		3(1)	4(2)	Z	-3.3	**0.001**
	2 n,%	7(17.9)	3(3.9)	X^2^	12.4	**0.006**
	3 n,%	14(35.9)	16(21.1)			
	4 n,%	13(33.3)	17(22.4)			
	5 n,%	5(12.8)	27(35.5)			
LEDD mg/day		600(655.0)	575(469)	Z	-0.7	0.460
Cognitive impairment n,%		10(25.6)	40(52.6)	X^2^	7.8	**0.020**
	Normal cognition n,%	29(74.4)	36(47.4)			
	PD-MCI n,%	7(17.9)	25(32.9)			
	PDD n,%	3(7.7)	15(19.7)			
Baseline CFS		5(2)	6(2)	Z	-4.6	**<0.001**
Schwab and England ADLs		70(20)	50(30)	Z	-4.4	**<0.001**
Total number of admissions		1(0)	1(2)	Z	-3.4	**0.001**
Total number of study visits		4.5(5)	6(8)	Z	-2.9	**0.004**

*Data are median and inter-quartile range (IQR) unless otherwise specified. Significant results highlighted in bold.*

* MDS-UPDRS III: n = *1 missing in PD no delirium group,* n = *14 missing in PD delirium group; † Hoehn and Yahr stage:* n = *13 missing in PD delirium group*

*PD* = *Parkinson’s disease; MDs-UPDRS III* = *Movement Disorders Society Unified Parkinson’s Disease Rating Scale Part III; LEDD* = *Levodopa Equivalent Daily Dose; PD-MCI* = *Parkinson’s disease with mild cognitive impairment; PDD* = *Parkinson’s disease dementia; CFS* = *Clinical Frailty Scale; ADLs* = *Activities of Daily Living.*

### Between group differences for bedside delirium tools

Differences in all bedside tool scores were compared in all cases with and without delirium (n = 200, Table 3). Significantly more cases with delirium (n = 122) scored positively on the delirium screening tools (4AT and CAM), scored higher for delirium severity (MDAS, DRS-98-R), were more confused (SQuiD), had more altered levels of arousal (GCS, OSLA and m-RASS) and demonstrated a poorer cognitive test performance (*P* < .01 for all) compared to those without delirium (n = 78).

**Table 3:** Between group differences in delirium cases with and without delirium.

	Tool	All admissions (n = 200)					Admission in those with cognitive impairment (n = 96)				
		PD no delirium n = 78 admissions	PD with delirium n = 122 admissions	Test statistic		p-value	PD no delirium n = 23 admissions	PD with delirium n = 73 admissions	Test statistic		p-value
	MDAS total (/30)	7(4)	18(11)	Z	-10.1	**<0.001**	11(5)	21(10)	Z	-5.3	**<0.001**
	4AT total (/12)	0.5(4)	8(7)	Z	-10.1	**<0.001**	3(4)	9(5)	Z	-5.4	**<0.001**
	CAM: yes n(%)	6(7.7)	105(86.1)	X^2^	120.3	**<0.001**	6(26)	67(91.7)	X^2^	41.4	**<0.001**
	DSR-98-R total (/39)	8(6)	21(10)	Z	-9.9	**<0.001**	12(6)	22(8.5)	Z	-5.6	**<0.001**
	SQuiD: yes n(%)	11(14.1)	81(66.4)	X^2^	53.3	**<0.001**	6(26.1)	55(75.3)	X^2^	18.3	**<0.001**
**Attention**	Digits forward (/5)	5(0)	4(5)	Z	-5.6	**<0.001**	5(2)	4(5)	Z	-1.9	0.057
	Digits backwards (/4)	3(4)	0(2)	Z	-5.5	**<0.001**	0(3)	0(0)	Z	-1.1	0.257
	MOTYB (/2)	0(1)	1(2)	Z	-5.2	**<0.001**	1(1)	1(2)	Z	-2.0	0.050
	SAVEAHAART: correct n(%)	43(55.1)	44(36.4)	X^2^	6.74	**0.009**	7(30.4)	22(30.6)	X^2^	0.0	0.991
	20to1: correct n(%)	56(71.8)	50(41.3)	X^2^	17.8	**<0.001**	11(47.8)	25(34.7)	X^2^	1.2	0.259
	Serial 7s (/5)	4(2)	1(2)	Z	-6.4	**<0.001**	2(3)	0(2)	Z	-2.5	**0.013**
	Trail Making Test Part A^*^ (seconds) (/300)	59(45)	300(156)	Z	-7.8	**<0.001**	85.5(95)	300(0)	Z	-4.4	**<0.001**
**Arousal**	GCS (/15)	15(1)	13(6)	Z	-6.6	**<0.001**	14(9)	12(6)	Z	-1.7	0.092
	OSLA (/15)	3(2)	7(7)	Z	-7.2	**<0.001**	4(4)	8(7)	Z	-3.5	**<0.001**
	m-RASS	0(0)	-1(4)	Z	-1.8	0.074	0(1)	-1(4)	Z	-0.1	0.927
	m-RASS modified scoring (/5)	0(1)	2(2)	Z	-8.2	**<0.001**	0(1)	2(2)	Z	-4.6	**<0.001**
**Cognitive tests**											
***Orientation***	Orientation total (/10)	8(3)	4(7)	Z	-8.2	**<0.001**	7(4)	3(6)	Z	-4.0	**<0.001**
	Age: correct n(%)	67(85.9)	72(59.5)	X^2^	16.5	**<0.001**	15(65.2)	36(50.0)	X^2^	1.6	0.203
***Memory***	Date of birth: correct n(%)	75(96.2)	79(65.3)	X^2^	27.4	**<0.001**	21(91.3)	44(61.1)	X^2^	7.4	**0.007**
	WWII end: correct n(%)	57(73.1)	55(45.5)	X^2^	15.2	**<0.001**	11(47.9)	28(38.9)	X^2^	0.6	0.448
	Immediate recall (/3)	3(0)	2.5(3)	Z	-4.8	**<0.001**	3(2)	2(3)	Z	-1.5	0.136
	Delayed recall (/3)	2(2)	0(2)	Z	-5.7	**<0.001**	1(2)	0(1)	Z	-1.7	0.081
***Visuospatial***	Stone float on water: correct n(%)	73(93.6)	77(63.6)	X^2^	24.2	**<0.001**	19(82.6)	41(56.9)	X^2^	4.9	**0.026**
***Language***	Object naming (/2)	2(0)	2(2)	Z	-5.3	**<0.001**	2(0)	2(2)	Z	-2.6	**0.010**
	Three stage command (/3)	3(0)	2(3)	Z	-6.4	**<0.001**	3(1)	1(3)	Z	-2.9	**0.003**

*Data presented are median and inter-quartile range (IQR) unless otherwise specified; Significant results after Benjamini-Hochberg procedure are highlighted in bold P ≤ .026;* *Missing data in n = 46 admissions. All admissions (n = 200) comprise n = 115 individual participants; Admission in those with cognitive impairment (n = 96) comprise n = 50 individual participants.

PD = Parkinson’s disease; MDAS: Memorial Delirium Assessment Scale, 4AT:4 As Test, CAM: Confusion Assessment Method, DSR-98-R: Delirium Rating Scale, SQuID: Single Question in Delirium; MOTYB: Months of the year backwards; GCS = Glasgow Coma Scale; OSLA = Observational Level of Arousal; m-RASS = Modified Richmond Agitation Scale; WWII: World War II.

In PD participants with known baseline cognitive impairment (PD-MCI and PDD), more cases with delirium (n = 73) scored positively on the delirium screening tools (4AT and CAM), scored higher for delirium severity (MDAS, DRS-98-R) and were more confused (SQuiD) compared to cases without delirium (n = 23, *P* < .001 for all). Of the arousal tools, the OSLA and m-RASS (*P* < .001 for both) indicated more altered levels of arousal in cases with delirium, but not GCS (*P* > .05). Only orientation (orientation total), long term memory (date of birth), visuospatial reasoning, attention (serial sevens, Trail Making Part A) and language (object naming and three stage command) were significantly impaired in participants with cognitive impairment and delirium compared to those without delirium (*P* ≤ .026 for all).

### Accuracy of delirium tools

**Figure 1:**
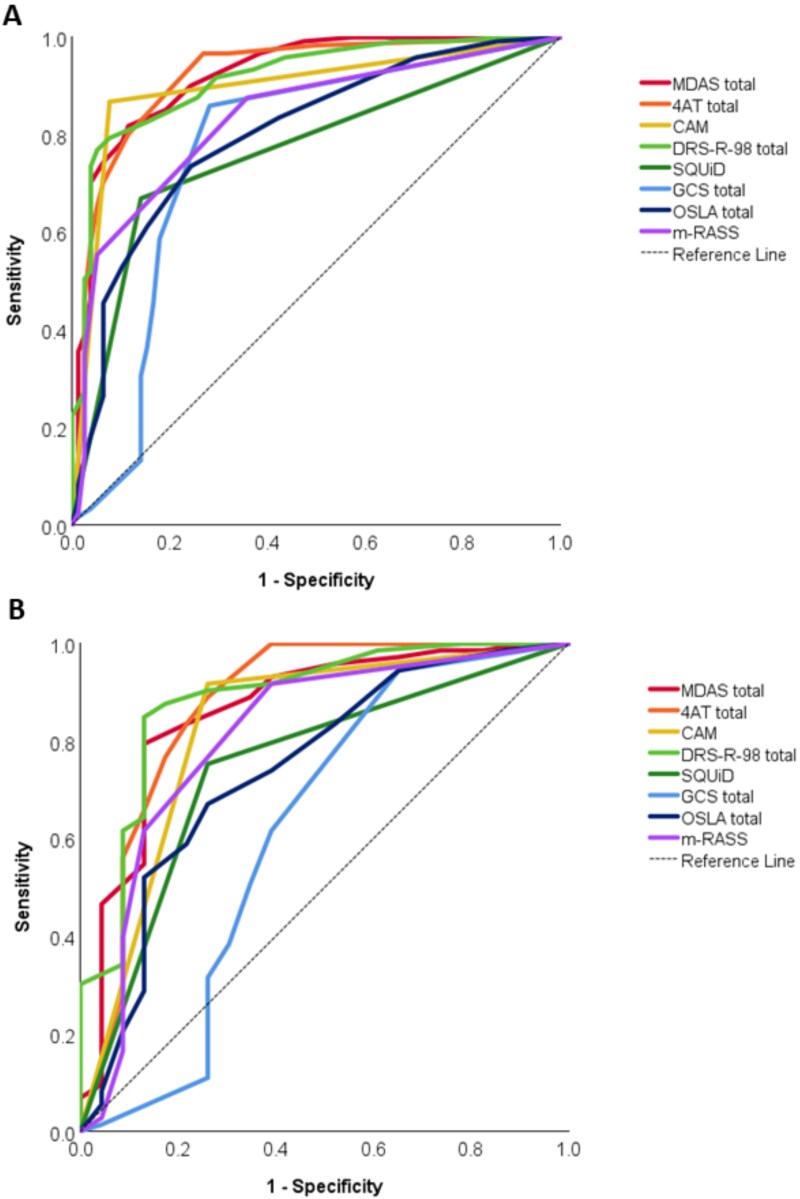
ROC curves for (A) all participant admissions and (B) participants with cognitive impairment. MDAS: Memorial Delirium Assessment Scale, 4AT:4 As Test, CAM: Confusion Assessment Method, DSR-98-R: Delirium Rating Scale, SQuID: Single Question in Delirium; GCS = Glasgow Coma Scale; OSLA = Observational Level of Arousal; m-RASS = Modified Richmond Agitation Scale.

The diagnostic accuracy of each delirium tool compared to the DSM-5 criteria were assessed using ROC curves (Table 3), firstly in all admissions (Figure 1A), and then in admissions in PD participants with known cognitive impairment (PD-MCI or PDD, Figure 1B). Paired ROC tests were used to determine accuracy between delirium tools (Supplementary Table 3).

Considering all participants, AUC ranged from 0.597–0.923 (*P* ≤ .032 for all, Table 4). Sensitivity ranged from 34.7-96.7%, while specificity ranged from 55.8-97.4%. Of the delirium tools, the MDAS total had the highest overall accuracy (AUC = 0.923, *P* < .001), followed by the 4AT (AUC = 0.922, *P* < .001). The 4AT had the best sensitivity (96.7%) but the poorest specificity (73.1%). The DSR-98-R had the best specificity (94.9%). The SQuiD had the poorest sensitivity (76.4%).

**Table 4:** Evaluating the accuracy and identifying optimal cut-off points for identifying delirium in PD participants with delirium and cognitive impairment.

Tool	All Participants										With cognitive impairment (PD-MCI and PDD)									
	AUC	p-value	95% CI		Optimal cut-off	Sens.	Spec.	Youden's index	PPV	NPV	AUC	p-value	95% CI		Optimal Cut-off	Sens.	Spec.	Youden's index	PPV	NPV
			Lower bound	Upper bound									Lower bound	Upper bound						
MDAS total	0.923	**<0.001**	0.88	0.96	≥12	0.818	0.885	0.703	0.92	0.76	0.864	**<0.001**	0.77	0.96	≥15	0.795	0.87	0.665	0.951	0.571
4AT total	0.922	**<0.001**	0.88	0.96	≥4	0.967	0.731	0.698	0.85	0.93	0.874	**<0.001**	0.77	0.98	≥5	0.89	0.739	0.629	0.915	0.68
CAM	0.895	**<0.001**	0.85	0.95	Yes	0.868	0.923	0.791	0.95	0.82	0.828	**<0.001**	0.72	0.94	Yes	0.918	0.739	0.657	0.918	0.739
DSR-98-R total	0.916	**<0.001**	0.88	0.96	≥15	0.769	0.949	0.718	0.96	0.73	0.886	**<0.001**	0.8	0.97	≥16	0.849	0.87	0.719	0.941	0.679
SQuID	0.764	**<0.001**	0.7	0.83	Yes	0.669	0.859	0.528	0.88	0.63	0.746	**<0.001**	0.63	0.87	Yes	0.753	0.739	0.492	0.944	0.476
**Attention**																				
Digits forward	0.715	**<0.001**	0.64	0.79	≤4	0.585	0.792	0.377	0.814	0.55	0.625	0.073	0.502	0.747	≤1	0.389	0.87	0.259	0.784	0.333
Digits backwards	0.706	**<0.001**	0.63	0.78	≤1	0.754	0.636	0.39	0.763	0.62	0.557	0.412	0.417	0.697	≤1	0.806	0.304	0.11	0.784	0.333
MOTYB	0.703	**<0.001**	0.63	0.78	>0	0.602	0.753	0.355	0.783	0.542	0.628	0.066	0.504	0.752	>1	0.356	0.87	0.226	0.897	0.299
SAVE A HEART	0.597	**0.022**	0.52	0.68	>1 error	0.636	0.558	0.194	0.688	0.494	0.499	0.993	0.363	0.636	>1 error	0.694	0.304	-0.002	0.759	0.242
20 to 1	0.652	**<0.001**	0.57	0.73	>1 error	0.576	0.727	0.303	0.767	0.528	0.566	0.346	0.429	0.702	>1 error	0.653	0.478	0.131	0.694	0.203
Serial 7s	0.765	**<0.001**	0.7	0.83	≤2	0.788	0.701	0.489	0.802	0.684	0.663	**0.019**	0.535	0.792	≤2	0.833	0.478	0.311	0.833	0.478
**Arousal**																				
GCS	0.767	**<0.001**	0.69	0.84	<15	0.86	0.718	0.578	0.83	0.77	0.615	0.098	0.46	0.77	<15	0.945	0.348	0.293	0.821	0.667
OSLA	0.802	**<0.001**	0.74	0.87	≥5	0.736	0.756	0.492	0.82	0.65	0.744	**<0.001**	0.62	0.87	≥6	0.671	0.739	0.41	0.891	0.415
m-RASS	0.834	**<0.001**	0.78	0.89	≥1	0.876	0.641	0.517	0.79	0.77	0.811	**<0.001**	0.69	0.93	≥1	0.918	0.609	0.527	0.882	0.7
**Cognition**																				
Orientation total	0.841	**<0.001**	0.79	0.9	≤6	0.686	0.831	0.517	0.863	0.627	0.775	**<0.001**	0.671	0.879	≤3	0.556	0.87	0.426	0.93	0.385
How old are you?	0.634	**0.002**	0.56	0.71	0	0.398	0.87	0.268	0.828	0.482	0.576	0.274	0.443	0.709	0	0.5	0.652	0.152	0.692	0.182
Date of birth	0.661	**<0.001**	0.59	0.74	0	0.347	0.974	0.321	0.953	0.487	0.651	**0.03**	0.533	0.769	0	0.389	0.913	0.302	0.677	0.067
WWII end?	0.641	**0.001**	0.56	0.72	0	0.542	0.74	0.282	0.765	0.509	0.545	0.52	0.408	0.681	0	0.611	0.478	0.089	0.718	0.214
Stone float on water	0.656	**<0.001**	0.58	0.73	0	0.364	0.948	0.312	0.915	0.487	0.628	0.065	0.504	0.752	0	0.431	0.826	0.257	0.683	0.114
Three-word immediate recall	0.677	**<0.001**	0.6	0.75	≤2	0.5	0.805	0.305	0.609	0	0.596	0.167	0.473	0.719	<1	0.361	0.913	0.274	0.929	0.309
Delayed recall	0.724	**<0.001**	0.65	0.8	<1	0.585	0.779	0.364	0.805	0.55	0.607	0.125	0.473	0.741	<1	0.653	0.565	0.218	0.825	0.342
Object naming	0.669	**<0.001**	0.59	0.74	≤1	0.373	0.961	0.334	0.936	0.497	0.649	**0.032**	0.531	0.768	≤1	0.417	0.87	0.287	0.909	0.323
Three stage command	0.735	**<0.001**	0.67	0.8	≤2	0.568	0.87	0.438	0.872	0.568	0.69	**0.006**	0.573	0.807	≤1	0.528	0.87	0.398	0.927	0.37

*Significant results after Benjamini-Hochberg procedure are highlighted in bold P ≤ .032. All admissions (*n = *200) comprise* n = *115 individual participants; Admission in those with cognitive impairment (*n = *96) comprise* n = *50 individual participants.*

MDAS: Memorial Delirium Assessment Scale, 4AT:4 As Test, CAM: Confusion Assessment Method, DSR-98-R: Delirium Rating Scale, SQuID: Single Question in Delirium; GCS: Glasgow Coma Scale; OSLA: Observational Level of Arousal; m-RASS: Modified Richmond Agitation Scale, MOTYB: Months of the year backwards; WWII: World War II; AUC: Area under the curve; CI = Confidence interval; Sens. = Sensitivity; Spec. = Specificity; PPV = Positive predictive value; NPV = Negative predictive value.

Of the arousal tools, m-RASS had the highest AUC (AUC = 0.834, *P* < .001) and the best sensitivity (87.6%). The OSLA had the best specificity (75.6%). Of the attention tools, Serial 7s had the highest AUC and sensitivity (AUC = 0.765, *P* < .001, sensitivity 78.8%), while digit span forward had the highest specificity (79.2%). The AUC of cognitive bedside tools ranged from AUC = 0.634-0.841; orientation total had the highest AUC and sensitivity (AUC = 0.841, *P* < .001, sensitivity 68.6%), followed by three-stage command (AUC = 0.735, *P* < .01, sensitivity 56.8%). However, date of birth had the highest specificity (97.4%) followed by object naming (96.1%) and visuospatial reasoning (stone float on water, 94.8%).

### Accuracy of delirium tools in those with known cognitive impairment

Repeating this analysis for those with known cognitive impairment (PD-MCI or PDD), only selected tools were significant, with a lower AUC compared to all participants (AUC = 0.499-0.886, Table 4). Sensitivity ranged from 35.6-94.5%, while specificity ranged from 30.4-91.3%. All the diagnostic/severity tools were significantly associated with delirium; the DSR-98-R total had the highest AUC (AUC = 0.886, *P* < .001) followed by the 4AT (AUC = 0.874, *P* < .001). The CAM had the highest sensitivity (91.8%) followed by the 4AT (89.0%).However, the optimal cut-offs for identifying delirium in those with PD and cognitive impairment were higher (more severe) compared the whole group.

Of the arousal tools, the m-RASS had the highest overall accuracy and high sensitivity (AUC = 0.811, *P* < .001, sensitivity 91.8%, specificity 60.9%) The GCS had the highest sensitivity (94.5%) while the OSLA had the highest specificity (73.9%). Serial 7s was the only significantly accurate attention test, although specificity was low (AUC = 0.663, *P* < .032, specificity 47.8%). Although not significant, the months of the years backward and forward digit span both had a high specificity of 87.0%. Of the bedside cognitive tools, only the three-stage command, object naming, date of birth and orientation tools were significantly associated with delirium (AUC = 0.645-0.775, *P* ≤ .032 for all). Although sensitivity was low, specificity was highest for date of birth (91.3%) followed by orientation total three-stage command and object naming (87% for all).

## Discussion

We found that current bedside tools can accurately identify delirium in PD inpatients. The MDAS and 4AT were the most accurate. However, in PD participants with known cognitive impairment, the accuracy of bedside tools dropped, and only selected tools of arousal and cognition were significantly associated with delirium. Furthermore, alternative cut-offs may be needed to detect delirium in PD inpatients with cognitive impairment.

It is of vital importance that delirium is accurately identified to reduce the risk of poor patient outcomes, including mortality and dementia [24]. Evaluating the accuracy of existing delirium bedside tools in PD is of particular importance due to overlapping phenotypic symptoms. Existing tools do not differentiate between acute changes in symptoms, which may be attributed to PD rather than delirium. This means that delirium could either be missed or incorrectly diagnosed [7, 9]. We found that in PD, commonly used bedside delirium tools were sensitive to, and able to accurately identify delirium in PD; this is consistent with previous studies in older adults [13, 19, 20, 25, 26]. We found that the MDAS and 4AT had the highest accuracy, followed by the DSR-98-R. We found that the 4AT had a comparable AUC to the MDAS, and a score of ≥4 had the highest sensitivity (97%) compared to the MDAS (82%) and the CAM (87%), suggesting it may be a good screening tool for delirium in PD. The MDAS is a commonly used delirium severity tool in research [27], most often used in inpatients with cancer and surgical settings [28, 29]. It has previously been shown to have good agreement with DSM criteria and the DRS-98-R in palliative care settings, comprising older adults with and without dementia [30], and good overall accuracy of 96% [31], which is comparable to 92% found in PD in our study. However, the MDAS can take 10-15 minutes to complete in addition to collecting collateral history [27].

A previous systematic review and meta-analysis in older adults with and without delirium reported that the 4AT performed well, with a pooled sensitivity of 88% and specificity of 88% [32], which included participants with dementia and/or stroke. The 4AT has also been reported to be more accurate than the CAM in older adults [25, 33] and in good agreement with DSM criteria, consistent with our findings. However, the 4AT is brief, requires no training, is already widely used in clinical practice and has been shown to perform well as a diagnostic tool [34]. As we found that the 4AT has comparable accuracy and high sensitivity to PD-delirium, we suggest that this would be an optimal screening tool for PD inpatients, with a score of ≥4 used as per guidance, but with a score of ≥5 in those with known cognitive impairment.

Of the brief bedside arousal and cognitive tools, we found that all tools were significantly impaired in admissions with delirium compared to those without. Of the arousal tools, the m-RASS had the highest sensitivity (87.6%), while the OSLA had the highest specificity (75.6%). We previously found that these assessments were accurate in Lewy body disease (LBD) delirium [13]. However, we previously found that the GCS was the most accurate in participants with LBD, while the OSLA was more sensitive to delirium in older adults without LBD or dementia. This may be due to the smaller numbers of only 69 admissions, compared to 200 admissions used in this analysis. Of the cognitive tools, impaired orientation was the most accurate tool followed by Serial 7s (attention) and three stage command (language). This is consistent with previous studies which reported that these tools were sensitive to delirium in older adults generally [14, 35, 36]. However, our results suggest that tools such as the m-RASS, orientation, Serial 7s and three-stage command may be specifically useful to aid the detection of delirium in PD.

We found that current delirium tools were less accurate in those with PD and known cognitive impairment, and that different cut-offs may be needed. This is consistent with our previous findings, albeit in a much smaller sample [13]. The accuracy ranged from 49.9% to 86.6%, with several tools not reaching significance. Consistent with previous studies in delirium superimposed on dementia (DSD), we found that DRS-98-R, 4AT and the MDAS could accurately identify delirium in PD-cognitive impairment [10, 25, 26]. However, the diagnostic accuracy differs between studies, possibly due to differences in protocols, populations and the proportion of participants with dementia being included, types of dementia included and severity of cognitive impairment [8, 29].

Of the arousal tools, the m-RASS and OSLA remained significant with high sensitivity, consistent with our findings in LBD [13], but not the GCS. This may be due to the large confidence intervals found in this study compared to our previous findings, indicating a more heterogenous presentation in this larger sample. Previous studies have also reported that the OSLA and m-RASS may be sensitive to DSD [37]. Only selective cognitive tools could accurately identify delirium in those with PD and known cognitive impairment in our study. Although this is consistent with previous findings in older adults with DSD [37] and LBD [13], it is perhaps unsurprising due to the overlapping presenting phenotype and baseline cognitive impairment [38]. DSD can be more challenging to identify, particularly in LBD [8, 38], thus accurate PD-specific tools to aid diagnosis are crucial to improving patient outcomes. We suggest that the tests identified in this study as being sensitive to delirium in those with known cognitive impairment could be used to derive a new PD-specific diagnostic tool. However, this would need to be validated in an independent cohort.

Strengths of this study include a large sample size using robust standardised procedures based on the DSM-5 criteria, collected prospectively. However, there were several limitations. Although this is the largest study of its kind to date, recruitment was limited by not being able to enter the hospital due to the COVID-19 pandemic. This was a secondary analysis as part of a prospective observational study, and bedside tools were not completed independently of, or blinded to, the reference standard (DSM-5 criteria). These were all performed during the same visit by the same researcher to reduce participant burden. This may also account for the higher than expected accuracy of the CAM, where sensitivity has previously been 40% in older adults [33]. To validate our exploratory findings, these tools and cut-points would need to be evaluated in a blinded manner. Therefore, the tools evaluated in this paper should not be considered in isolation to diagnosis delirium, but alongside clinical judgment. To evaluate the bedside tools in those with cognitive impairment, we repeated our analysis in those with known PD-MCI or PDD prior to hospital admission. While some participants may have had undiagnosed cognitive impairment, we mitigated this by including all participants regardless of cognitive status in the main analysis.

We took a pragmatic approach and used the MDAS total to identify the study visit with peak delirium severity for this analysis. We acknowledged that this may influence the accuracy of tools, including the MDAS. However, this was mitigated by the MDAS total not being used as part of the DSM-5 criteria. Furthermore, this analysis was cross-sectional and did not consider the fluctuating nature of delirium over the course of the admissions and should be considered by future studies. Finally, we compared existing tools to the DSM-5 criteria as our reference standard. We recognise that, in the future, this may change as criteria are updated. Although replication may be needed when future criteria are published, our findings provide a basis for future studies.

In summary, current bedside tools can accurately identify delirium in PD inpatients. Although the MDAS and 4AT were comparable, the 4AT may have better clinical utility as a screening tool with higher sensitivity and diagnostic accuracy; it is also quicker to complete and is already in widespread clinical use. Delirium tools and selective bedside tools may also help to identify delirium in those with PD-MCI or PDD, but alternative cut-offs may be required (e.g. 4AT ≥5 for those with known baseline cognitive impairment, and ≥4 otherwise). However, caution is recommended as tools did not specifically differentiate between PD features and acute symptoms associated with delirium, and bedside tools were not completed independently to the DSM-5 criteria. A PD specific delirium tool may be helpful for future delirium management and clinical trials.

## Supplementary Material

aa-25-0382-File002_afaf197

## Research Data Transparency and Availability

Unidentifiable data may be shared on request to the corresponding author.
