# Supplementary material for: Detecting delirium in Parkinson’s disease: an evaluation of diagnostic accuracy of bedside tools
Source: Age Ageing. 2025 Jul 20;54(7):afaf197. doi: 10.1093/ageing/afaf197 (PMC12276198; doi:10.1093/ageing/afaf197)
Supplement: aa-25-0382-File002_afaf197 [file aa-25-0382-file002_afaf197.docx]

Appendix: Supplementary data

**Supplementary Table 1 – Operationalised DSM-5 diagnostic criteria from the DECIDE study**

| **DSM-5 criteria** | **Test to be performed or information needed** |
| --- | --- |
| A. Disturbance in attention (i.e., reduced ability to direct, focus, sustain, and shift attention) and awareness (reduced orientation to the environment). | Observations by the examiner during the interview (initiated by questioning such as “can you tell me what has been going on today?”)  Level of arousal measured using m-RASS and OSLA  Months of the year backwards  Digit Span from MDAS |
| B. The disturbance develops over a short period of time (usually hours to a few days), represents a change from baseline attention and awareness, and tends to fluctuate in severity during the course of a day. | Acute onset and/or fluctuation obtained from informant history from nursing staff, next of kin and clinical notes |
| C. An additional disturbance in cognition (e.g., memory deficit, disorientation, language, visuospatial ability, or perception). | Impairment in any of the following domains:  SHORT-TERM MEMORY: three item recall at three minutes LONG-TERM MEMORY: when did World War II end?  ORIENTATION: 10 orientation questions from MDAS  LANGUAGE: 3 stage command, naming an object and explain purpose of object along with fluency, comprehension, and content of conversation  VISUOSPATIAL: Will a stone float on water?  PERCEPTUAL DISTURBANCE: evidence of illusions or hallucinations by collateral or direct observation/questioning |
| D. The disturbances in criteria A and C are not explained by another pre-existing, established, or evolving neurocognitive disorder and do not occur in the context of a severely reduced level of arousal, such as coma. | Information from history/chart/clinical examination |
| E. There is evidence from the history, physical examination, or laboratory findings that the disturbance is a direct physiologic consequence of another medical condition, substance intoxication or withdrawal (i.e., because of a drug of abuse or to a medication), or exposure to a toxin or is because of multiple aetiologies. | Information from history/chart/clinical examination |

Delirium and Cognitive Impact in Dementia (DECIDE) [1]; Modified-Richmond Agitation and Sedation Scale – m-rass; Observational Scale of Level of Arousal – OSLA; Memoria Delirium Assessment Scale – MDAS; Diagnostic and Statistical Manual of Mental Disorders, Fifth Edition – DSM-5

1 Richardson SJ, Davis DHJ, Stephan BCM, et al. Recurrent delirium over 12 months predicts dementia: results of the Delirium and Cognitive Impact in Dementia (DECIDE) study. *Age Ageing* 2021;50:914-20.

Supplementary Table 2: Operationalisation of the Confusion Assessment Method (CAM)

*The diagnosis of delirium by CAM requires the presence of features 1* ***and*** *2 and* ***either*** *3 or 4.*

| **Feature** | **Description** | **Tick if feature present** |
| --- | --- | --- |
| *[1] Acute Onset and Fluctuating Course* | This feature is usually obtained from a family member or nurse and is shown by positive  responses to the following questions:   - Is there evidence of an acute change in mental status from the patient's baseline? - Did the (abnormal) behaviour fluctuate during the day, that is, tend to come and go, or increase and decrease in severity? |  |
| *[2] Inattention* | This feature is shown by a positive response to the following question:   - Did the patient have difficulty focusing attention, for example, being easily distractible, or having difficulty keeping track of what was being said? |  |
| *[3] Disorganized Thinking* | This feature is shown by a positive response to the following question:   - Was the patient's thinking disorganized or incoherent, such as rambling or irrelevant conversation, unclear or illogical flow of ideas, or unpredictable switching from subject to subject? |  |
| *[4] Altered Level of Consciousness* | This feature is shown by any answer other than "alert" to the following question:   - Overall, how would you rate this patient's level of consciousness?   Alert [normal]  Vigilant [hyper alert]  Lethargic [drowsy, easily aroused]  Stupor [difficult to arouse]  Coma [unarousable] |  |

**Delirium? Yes / No**

Supplementary Figure 1: Flow diagram of recruitment


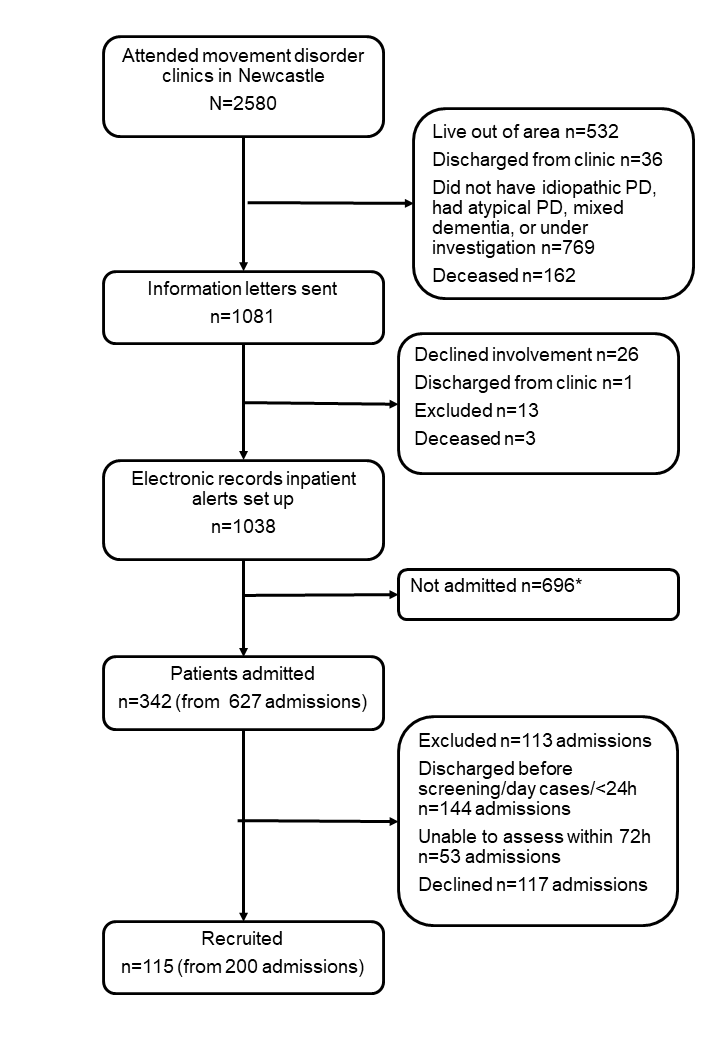


PD = Parkinson’s disease; *Does not include people with PD admitted to hospital during study pauses due to COVID-19 pandemic

**Supplementary Table 3: Comparison of paired receiver operating curves (ROC)**

| **Domain** | **Test/tool** | **MDAS total** | **4AT total** | **CAM** | **DSR-98-R total** |  |  |  |  |
| --- | --- | --- | --- | --- | --- | --- | --- | --- | --- |
|  | ***4AT total*** | -0.1 |  |  |  |  |  |  |  |
|  | ***CAM*** | -1.2 | 1.3 |  |  |  |  |  |  |
|  | ***DSR-98-R total*** | -0.7 | 0.4 | -0.9 |  |  |  |  |  |
|  | ***SQuiD*** | **4.8***** | **4.6***** | **3.9***** | **4.6***** |  |  |  |  |
| **Attention** |  | **Digits forward** | **Digits backwards** | **MOTYB** | **SAVEAHEART** | **20to1** |  |  |  |
|  | ***Digits backwards*** | 0.5 |  |  |  |  |  |  |  |
|  | ***MOTYB*** | **2.6*** | 1 |  |  |  |  |  |  |
|  | ***SAVEAHEART*** | **3.1**** | **2.3*** | 1.8 |  |  |  |  |  |
|  | ***20to1*** | **2.3*** | 1.6 | 1 | -0.8 |  |  |  |  |
|  | ***Serial 7s*** | 0.1 | -0.3 | -1.6 | **-2.9**** | **-2.1*** |  |  |  |
| **Arousal** |  | **GCS** | **OSLA** |  |  |  |  |  |  |
|  | ***OSLA*** | -0.8 |  |  |  |  |  |  |  |
|  | ***m-RASS*** | -1.6 | -1.1 |  |  |  |  |  |  |
| **Cognitive tests** |  | **Orientation total** | **Age** | **Date of birth** | **WWII end** | **Immediate recall** | **Delayed recall** | **Stone float on water** | **Object naming** |
| ***Orientation*** | ***Age*** | **6.8***** |  |  |  |  |  |  |  |
| ***Memory*** | ***Date of birth*** | **6.3***** | 0.9 |  |  |  |  |  |  |
|  | ***WWII end*** | **6.2***** | -0.1 | 0.6 |  |  |  |  |  |
|  | ***Immediate recall*** | **5.7***** | -1.3 | -0.6 | -1.0 |  |  |  |  |
|  | ***Delayed recall*** | **3.4**** | **-2.5*** | -1.8 | **-2.1*** | -1.3 |  |  |  |
| ***Visuospatial*** | ***Stone float on water*** | **6.7***** | -0.6 | 0.2 | -0.5 | 0.8 | **2.0*** |  |  |
| ***Language*** | ***Object naming*** | **6.2***** | -1.2 | -0.4 | -0.8 | 0.3 | 1.7 | -0.7 |  |
|  | ***Three stage command*** | **3.6***** | **-3.3**** | **-2.9**** | **-2.7**** | **-2.2*** | -0.3 | **-3.4** | **-3.0**** |

Data presented are effect size (z); significant results are highlighted in bold. *p<0.05; **p<0.01; ***p<0.001.

MDAS: Memorial Delirium Assessment Scale, 4AT:4 As Test, CAM: Confusion Assessment Method, DSR-98-R: Delirium Rating Scale, SQuID: Single Question in Delirium; GCS= Glasgow Coma Scale; OSLA= Observational Level of Arousal; m-RASS= Modified Richmond Agitation Scale, WWII: World War II.
